# Efficacy of a Combined Periarticular and Intraosseous Multimodal Analgesic Injection Technique in Simultaneous Bilateral Total Knee Arthroplasty: A Randomized Controlled Trial

**DOI:** 10.7759/cureus.53946

**Published:** 2024-02-09

**Authors:** Patcharavit Ploynumpon, Vajara Wilairatana, Thakrit Chompoosang

**Affiliations:** 1 Orthopedic Surgery, Rajavithi Hospital, Bangkok, THA; 2 Orthopedic Surgery, Chulalongkorn University, Bangkok, THA

**Keywords:** multimodal analgesia, postoperative pain, intraosseous injection, periarticular injection, total knee arthroplasty

## Abstract

Introduction

Early postoperative pain poses a challenge for surgeons to manage after total knee arthroplasty (TKA). Various techniques have been employed to optimize pain reduction, including Periarticular Multimodal Analgesia (PMA), recognized as a safe and effective method. Our study aims to enhance PMA through a combined intraosseous injection (PMA-I) and compare it with standard PMA.

Methods

Forty patients undergoing simultaneous bilateral TKA surgery were enrolled. Patients were randomized to receive PMA-I on one side of the knee, while the contralateral knee received standard PMA. Pain scores, bleeding, and range of motion (ROM) were assessed in both groups.

Results

The PMA-I group demonstrated statistically significant lower visual analog scale (VAS) scores at all postoperative time points, except at 48 hours, where the difference was not statistically significant. Postoperative bleeding and ROM did not significantly differ between groups.

Conclusion

PMA-I demonstrated both statistically and clinically significant reduction in early post-TKA pain, without additional costs, providing a technique that can be used to optimize postoperative pain control in TKA.

## Introduction

Patients in the advanced stages of osteoarthritis, for whom conventional treatments fail to alleviate knee pain, may find relief through total knee arthroplasty (TKA), a procedure known to enhance overall quality of life [1,2]. In spite of the overall success of TKA, a significant number of recipients, accounting for 60%, report experiencing severe postoperative knee pain, while 30% endure moderate pain. The acute postoperative pain associated with TKA has instilled fear in some patients, leading them to either delay or completely avoid undergoing the procedure [3]. Notably, up to 20% express dissatisfaction post-surgery, primarily attributing it to persistent postoperative pain [4]. Traditionally, opioids have been widely employed for perioperative pain management in surgical patients. However, the escalating synthetic opioid-related overdose deaths in recent years have prompted a reevaluation of opioid-only postoperative analgesic protocols, already under scrutiny for associated adverse effects such as drowsiness, fatigue, confusion, delirium, nausea, vomiting, ileus, and urinary retention, which hinder a swift functional recovery after surgery [5].

While monotherapy with opioids falls short in providing satisfactory postoperative pain relief after TKA, it remains prevalent in pain management. Various strategies have been explored to alleviate this pain, and Enhanced Recovery After Surgery (ERAS) has emerged as a recent protocol aimed at reducing hospital stays and improving patient satisfaction [6]. Employing a multimodal analgesic approach, ERAS integrates interventions such as femoral nerve blocks, epidural nerve blocks, and oral or injected pain medications, encompassing both oral analgesia and intravenous opioids [7-9]. Nonetheless, these procedures are associated with complications such as blocking failure, urinary retention, and systemic opioid-related adverse effects [10].

Periarticular multimodal analgesia (PMA) injection is utilized to enhance postoperative pain management by reducing local inflammatory responses following TKA. Numerous studies have explored the advantages of periarticular drug infiltration. In a systematic review conducted by Jiang et al., it was demonstrated that infiltration of analgesics for pain control provides superior pain relief compared to exclusive intravenous analgesia [11]. Another study by Koh et al. observed that pain reduction and functional outcomes were evident for up to 48 hours after the surgery [12].

Despite its use, the optimal location and formulation of the mixture remain inconclusive and subject to ongoing study. A retrospective study found that patients receiving intraosseous morphine through a catheter post-TKA exhibited significantly lower postoperative pain compared to the control group [13]. In an effort to further reduce local inflammatory responses, we hypothesize that a combined periarticular and intraosseous multimodal injection (PMD-I) technique could more effectively alleviate postoperative pain than PMD alone, without increasing costs or incorporating additional techniques. The aims of our study are to provide an alternative injection technique to the standard method without additional surgical steps or costs.

## Materials and methods

This was a randomized, double-blinded controlled trial study conducted in a single institution. The study protocol was approved by the Institutional Ethical Board Committee and all subjects were required to give written informed consent before participating (1555/2023).

All patients who underwent simultaneous bilateral primary TKA were enrolled, but those with a history of allergy to intervention medicine for abnormal coagulopathy (INR > 1.4 or aPTT ratio > 1.4), ischemic heart disease, or previous knee surgery were excluded. The patients were randomly allocated to intervention and control groups using a computer-generated block of four randomizations and opaque sealed envelopes. The staff involved in the clinical care, as well as the patients and the assessors, were unaware of the treatment assignments.

During surgery, the sealed envelopes were opened by the operation room staff, who were not involved in the study and who prepared both intraosseous and periarticular solutions of the same volume and appearance in order to blind the patients and surgeon.

Bilateral total knee replacement surgery was performed using a mid-vastus approach using a tibial first technique with a cemented posterior stabilized prosthesis (Stylers, USA) under spinal anesthesia by a single experienced surgeon who had performed more than 200 total knee replacements per year. On the periarticular plus intraosseous side, the intraosseous mixture solution (consisting of 15 mg ketorolac (0.5mL) and 500 mg of tranexamic acid (10mL), was injected into the medullary canal via the bone-cutting site to prepare to insert a prosthetic joint with the hole closed using the bone plug (the remaining blood clot in the intramedullary canal will be removed to giving space for mixture solution). The periarticular area was injected with 50mg of 0.5% bupivacaine (0.5% Marcaine,10mL) with epinephrine (1mg/mL) 0.1mL (equaling 100mcg).

On the periarticular side, 25mL of normal saline was injected into the femoral medullary canal while the periarticular area was injected with the periarticular mixture solution consisting of 15 mg ketorolac (0.5mL), 500 mg of transamine acid (10mL), 50mg of 0.5% bupivacaine (0.5% Marcaine, 10mL), and epinephrine (1 mg/mL) 0.1mL (equal to 100mcg).

**Figure 1:**
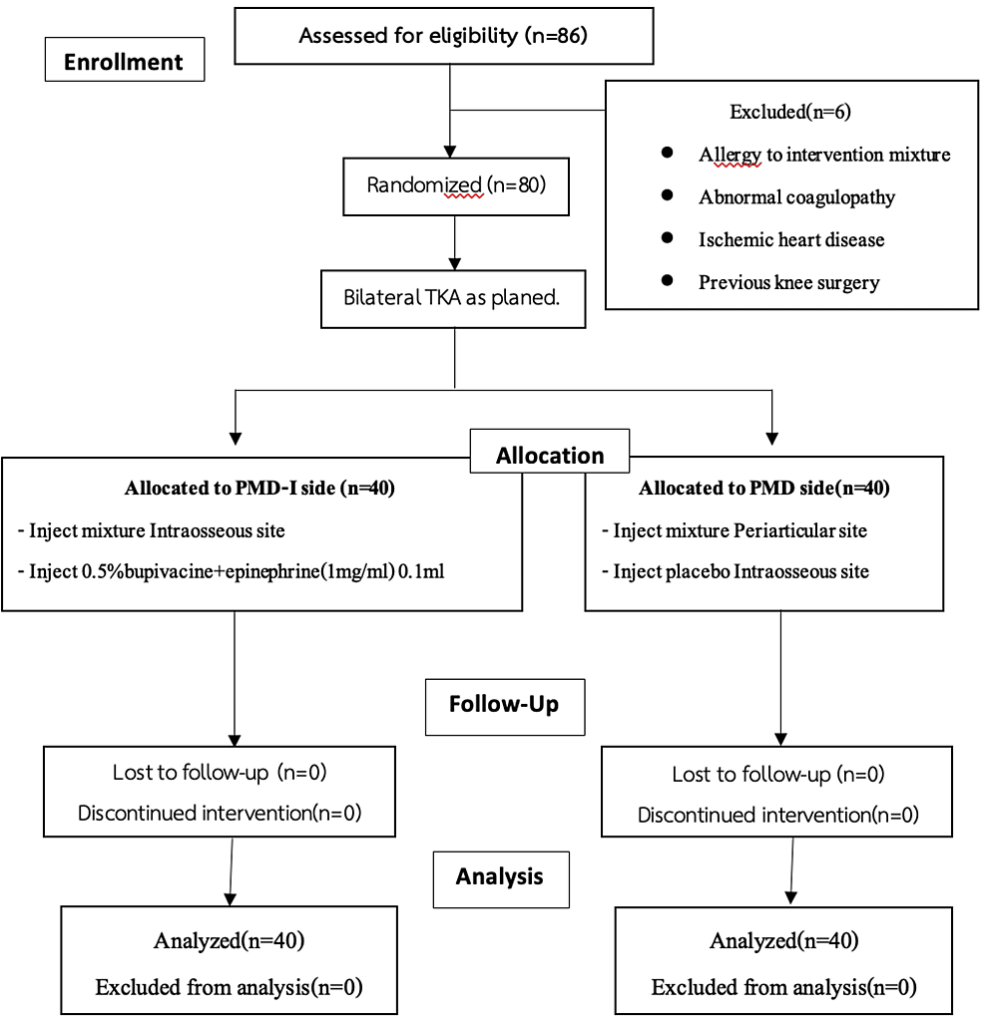
Study flowchart PMD, periarticular multimodal drug injection; PMD-I, Combined intraosseous and periarticular multimodal drug injection.

The same postoperative pain management protocol (comprising Naproxen 250 mg oral twice daily, Paracetamol 500 mg q 4 hrs, Tizanidine (4 mg) 2 oral q 6 hrs, and Gabapentin (100 mg q 8 hrs) was given to patients in each group, all of whom were allowed to ambulate the day after the surgery. Visual analog scale (VAS) was recorded at days 0 (12 hours post-operatively), 1, 2, 3, and two weeks after surgery. Range of motion (ROM) and postoperative bleeding were also recorded from the continuous passive motion device and the drain bottle (Figure 1).

Descriptive statistics were used to summarize the baseline characteristics of the patients. The normality of data distribution was assessed for continuous variables using the Shapiro-Wilk test and histograms. Student's t-test was used for between-group comparisons of means for normally distributed data, and the Mann-Whitney U test was employed for non-parametric data, while the Chi-squared test and Fisher’s exact test were used for categorical data analysis. Two-way repeated measures analysis of variance was used for between-group comparisons of VAS pain score over time using ANOVA and linear mixed models for normally distributed and non-parametric data respectively. The results were analyzed using SPSS software (Statistical Package for Social Sciences, version 22.0; IBM Corp., Armonk, NY) with statistical significance set at P < 0.05.

## Results

In our study, a total of 40 patients with osteoarthritis of both knees underwent simultaneous bilateral TKA, making a total of 80 knees. The demographic characteristics of the patients are summarized in Table 1.

**Table 1:** Demographic data PMD-I; combined intraosseous and periarticular injection, PMD; periarticular injection

	PMD-I	PMD	P-value
No. of patients	40	40	
Side of knee (intervention: control)	40	40	
Mean age (years)	67 +/- 6	64 +/- 5.4	0.79
Gender (Male: Female)	1:39		
Mean length of stay (days)	5.4 +/-1.08		
Knee severity score (Kellgren-Lawrence Grading)	Grade 3 :14 (35%)	Grade 3 :20 (50%)	0.18
	Grade 4 :26 (65%)	Grade 4 :20 (50%)	

**Figure 2:**
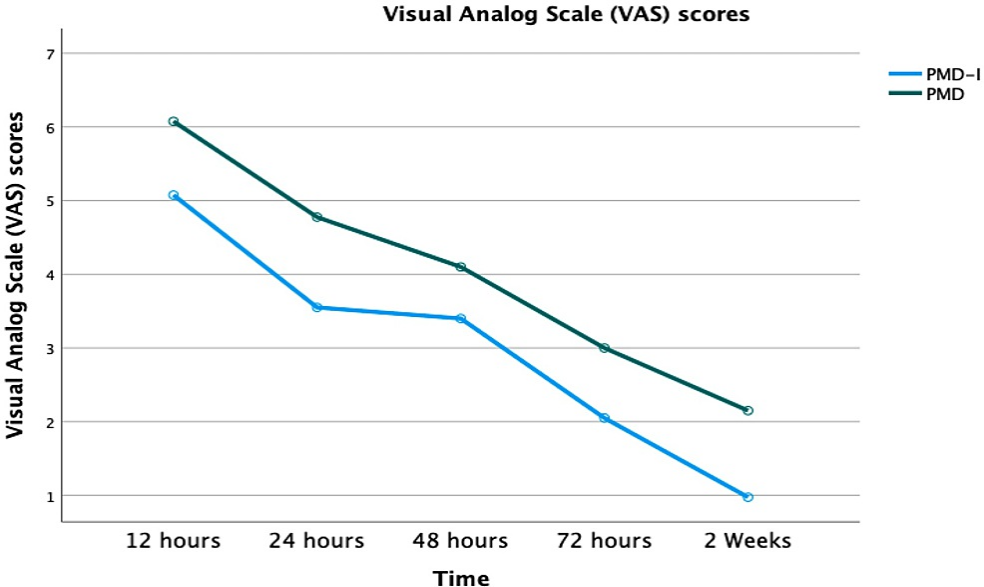
Visual analog scale (VAS) scores change compared both treatment groups PMD, periarticular multimodal drug injection; PMD-I, Combined intraosseous and periarticular multimodal drug injection

In the intraosseous plus periarticular injection group, the overall VAS score was 3.01 (95% CI: 2.719-3.301). At six hours post-operatively, it was 5.075 (95% CI: 4.56-5.5), while at 24 hours it was 3.55 (95% CI: 3.06-4.03), at 48 hours it was 3.4 (95% CI: 2.93-3.8), and at 72 hours it was 2.05 (95% CI: 1.73-2.36), lastly at 2 weeks, it was 0.9 (95% CI: 0.74-1.2). In the periarticular injection group, the overall VAS score was 4.02 (95% CI: 3.72-4.31): 6.07 (95% CI: 5.5-6.5), 4.7 (95% CI: 4.28-5.2), 4.1 (95% CI: 3.6-4.56), 3.0 (95% CI: 2.68-3.3) and 2.1(95% CI: 1.9-2.3) at 6, 24, 48, 72 hours and two weeks, respectively) (Figure 2).

The intraosseous plus periarticular multimodal injection group exhibited statistically significantly lower VAS scores than their periarticular multimodal injection counterparts as the results were summarized in Table 2. Additionally, there were no alterations in vital sign status during the intra-operative injection in both groups.

**Table 2:** Summary of all result in both treatment groups ROM, Range of motion; PMD, periarticular multimodal drug injection; PMD-I, Combined intraosseous and periarticular multimodal drug injection; SD, standard deviation; VAS, visual analog scale; Mean diff, Mean different between groups. Statistical significance (P < 0.05)

Outcome	PMD-I (Mean ± SD)	PMD (Mean ± SD)	Mean Diff.	P-value
VAS score				
12 hours	5.07 ± 1.67	6.07 ± 1.57	-1	.007
24 hours	3.55 ± 1.46	4.7 ± 1.62	-1.22	< .001
48 hours	3.4 ± 1.61	4.1 ± 1.29	-0.7	.36
72 hours	2.05 ± 1.08	3 ± 0.9	-0.95	< .001
2 weeks	0.9 ± 0.7	2.1± 0.7	-1.175	< .001
Drainage blood loss(mL)				
24 hours	122 ± 35	139 ± 53.9	-17.3	.092
48 hours	117 ± 57.9	135 ± 65.5	-17.25	.216
Degree of ROM				
24 hours	66.7 ± 13.4	63.5 ± 14.6	3.2	.29
48 hours	77.7 ± 9.4	76 ± 13.3	1.7	.5

Post-operative drainage blood loss

**Figure 3:**
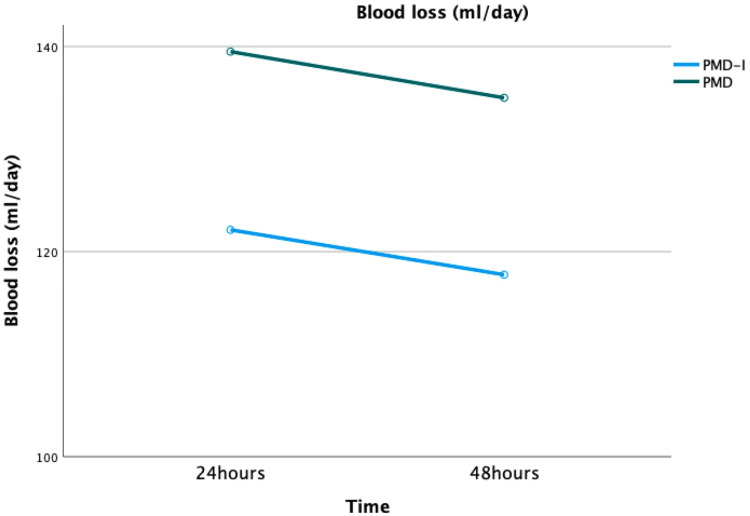
Post-operative blood loss compared both treatment groups PMD, periarticular multimodal drug injection; PMD-I, Combined intraosseous and periarticular multimodal drug injection

The mean bleeding per drain up to 48 hours was 119 ml/day (95% CI: 105.6-134.2) for the intraosseous plus periarticular injection group and 137.2 ml/day (95% CI: 123-151) in the periarticular injection group. The difference between the amount of bleeding in the two groups was not statistically significant (P=0.092) (Figure 3).

Range of motion

The overall mean ROM up to 48 hours was 72 degrees (95% CI: 68.6-75.8) in the intraosseous plus periarticular injection group and 69.7 degrees (95% CI:66.1-73.3) for the periarticular injection group, and this was without statistical significance (P=0.325).

## Discussion

With regard to PMD, the surgeon injects the mixture of components before suturing the surgical wound at the thigh (quadriceps) muscle fascia in front of the knee, joint capsule, and collateral ligament. There is no consensus on the formula for the mixture; however, many studies have used a combination regimen containing long-acting analgesia, intravenous NSAID, TXA, and vasopressor drugs [14,15].

In this RCT, we compared the efficacy of postoperative pain management with a PMD-I with that of the use of periarticular alone in the same simultaneous bilateral knee patients. Our study revealed that the PMD-I technique resulted in a significantly lower level of postoperative pain than that experienced by those who received only the PMD. This difference was observed at all postoperative time points except at the 48-hour time point.

The significantly better results witnessed in the PMD-I group may potentially be attributable to the localized absorption and localized reducing the inflammatory response of the analgesic cocktail mixture. In a previous study by Brozovich et al., morphine was injected into the femur via intraosseous infusion using a catheter, and it was found that the group of patients receiving morphine injections after knee surgery had statistically significant lower postoperative pain, without an increase in side effects related to morphine use than that experienced by the control group [13]. Also, the study of Ali Akın Ugras found the rising of local intraarticular inflammatory marker (IL-6) in TKA patients correlated with slower recovery but not correlated with a systemic inflammatory marker which suggests that the early recovery was affected by the local inflammatory response than the systemic responds [16].

Although the pain reduction in the PMD-I side reached statistical significance (mean difference = -0.86, P=0.05, 95% CI: -1.73 to -0.02), this technique alone may achieve clinical significance only at 12 hours, 24 hours, and two-week time points. A study by Myles et al. [17] reported that the minimal clinically important difference for acute postoperative pain is 10 of 100mm of VAS score. In conclusion, considering its minimal alteration from the standard method and its lack of additional cost, our technique can potentially be used as a supplemental injection technique to optimize postoperative pain control in TKA patients.

While previous studies have demonstrated improved functional outcomes following PMD injection [18,19], our study found no significant difference in range of motion (ROM) and overall functional outcome when utilizing the PMD-I technique. The improvements observed in prior studies may be attributed to PMD injection techniques targeting periarticular muscles and ligaments. However, this effect does not extend to intraosseous injection.

The addition of TXA to periarticular infiltration has been proposed as a means to reduce postoperative bleeding by acting as a competitive inhibitor of plasminogen, a crucial component in the fibrinolytic pathway essential for hemostasis. TXA can be administered intraarticularly or during intraoperative washing [20]. Peng et al. demonstrated a noteworthy reduction in hidden blood loss (HBL) and overall blood loss in the TXA group receiving periarticular infiltration compared to intravenous TXA administration [21]. Kim et al. reported decreased bleeding when periarticular infiltration and intravenous administration of TXA were combined [22]. In theory, direct injection of TXA into the medullary canal decreases blood loss, but at the same time, it increases systemic absorption. However, our study did not reveal a statistically significant difference in postoperative blood loss between the groups. Consequently, we cannot conclusively assert that employing the PMD-I technique will provide superior benefits over PMD alone in terms of blood loss.

The main strength of our research lies in the fact that there have been no previous prospective studies directly comparing the efficacy of PMD-I and PMD alone. In addition, our study was performed by a single surgeon in a single center using the same midvastus approach, technique, and prothesis design, which should have helped minimize variations in surgical techniques. A previous meta-analysis by Liu et al. compared different approaches (midvastus, subvastus) and found that the medial peripatellar approach pain score in the midvastus group was significantly lower than in the subvastus group at two weeks postoperatively, and that ROM was significantly higher at one week postoperatively [23].

The main limitation of our research was the strong female predominance in the study group, which is probably due to the tendency for Asian females to have sedentary lifestyles and high BMI [24]. A study by Matilde et al. found that women reported significantly higher postoperative pain after TKA than their male counterparts, and the influence of our demographic data therefore has to be taken into consideration. Secondly, we performed intraosseous injection only in the femur because this technique uses the intramedullary guide, which gives us access to the intramedullary canal of the femur, while on the tibial side, there was no medullary access because of our use of the extramedullary guide technique for the bone cut. Finally, we were not able to directly compare the intraosseous and PMD groups, and this may have resulted in the positive outcomes of the study being more pronounced due to the potential cardiovascular side effect of the bupivacaine and epinephrine intraosseous route injection [25,26]; however, we strived to make them as comparable as possible in order to minimize confounding variables and enhance the validity of our experimental design.

## Conclusions

The PMD-I demonstrates a significant reduction in postoperative pain compared to Standard PMD with minimal alteration from the standard method and the absence of added costs. Despite limitations, including a predominantly female study group, our study suggests that the intraosseous plus periarticular technique could serve as a supplemental approach for optimizing postoperative pain control in TKA.
